# E-liquid exposure induces bladder cancer cells to release extracellular vesicles that promote non-malignant urothelial cell transformation

**DOI:** 10.1038/s41598-022-27165-z

**Published:** 2023-01-04

**Authors:** Ryan D. Molony, Chia-Hao Wu, Yi-Fen Lee

**Affiliations:** 1 Department of Urology, School of Medicine and Dentistry, University of Rochester Medical Center, University of Rochester, 601 Elmwood Ave, Box 656, Rochester, NY 14642 USA; 2 Wilmot Cancer Center, University of Rochester, Rochester, USA; 3 Department of Pathology, University of Rochester, Rochester, USA

**Keywords:** Cancer, Molecular biology, Diseases, Medical research, Molecular medicine, Oncology, Risk factors

## Abstract

The vaping of electronic cigarettes (E-cigarettes) has recently emerged as a popular alternative to traditional cigarette smoking, but its association with bladder cancer (BC) risk remains to be established. BC patients exhibit high rates of recurrent disease, possibly as a consequence of the field cancerization effect. We have shown that BC-derived extracellular vesicles (BCEVs) can permanently alter recipient urothelial cells in predisposed fields such that they become fully transformed malignant cells. To model the role that BCEVs may play in this potentially oncogenic setting, we treated TCCSUP BC cells with cigarette smoke extract, unflavored E-liquid, or menthol flavored E-liquid. Those treated BCEVs were then tested for their tumorigenic potential. We found that these smoking- and E-cigarette-related BCEVs were able to promote oxidative stress, inflammatory signaling, and DNA damage in recipient SV-HUC urothelial cells. Strikingly, menthol E-liquid-induced BCEVs significantly increased rates of malignant urothelial cell transformation. While further in vivo validation of the simultaneous effects of E-liquid and E-liquid-induced BCEVs on field cancerization is needed, these data highlight the possibility that E-cigarettes may compound user risk in a manner that can contribute to higher rates of BC incidence or recurrence.

## Introduction

Bladder cancer (BC) is the ninth most common form of cancer and the thirteenth leading cause of cancer-related death^1^. The majority of newly diagnosed BC patients have non-muscle invasive (NMI) BC^2,3^. Two-thirds of BC patients will develop tumor recurrence within 5 years, and 88% will experience recurrent disease within 15 years^4^. This imposes an enormous physical, psychological, and economic burden on affected patients, as a BC diagnosis necessitates a lifetime of surveillance to detect and treat tumors as they reemerge resulting in the highest lifetime per-patient treatment costs of any cancer type^5^. BC is heterogeneous, and multiple coexisting tumors often arise within the same patient. The processes that govern such tumor heterogeneity and recurrence may be in part driven by the field cancerization effect, which refers to the development of a pre-malignant region that is predisposed to subsequent tumor formation through mutational accumulation^6,7^. To date, this effect has been reported in various cancers, including BC^8^. This pre-malignant field may pose a higher risk of BC recurrence, and the tumors that consequently arise may or may not share similar histology with the primary tumors^9^, thus making it difficult to predict prognostic outcomes for these secondary tumors and posing challenges for their clinical management. Further efforts to understand the mechanistic drivers of this BC field cancerization effect are thus needed to aid in the design of appropriate molecular diagnostic techniques suitable for the early detection and identification of precursor lesions capable of decreasing morbidity and mortality.

Cigarette smoking is the best-established risk factor associated with BC development^10,11^, with smokers exhibiting a threefold increase in their odds of being diagnosed with this form of cancer^12^. Roughly half of all BC cases are potentially attributable to smoking, and recent evidence suggests that in addition to increasing the risk of BC incidence, smoking can also contribute to higher recurrence and mortality rates in patients with both NMIBC and MIBC^13,14^. Tobacco contains over 60 carcinogens that can contribute to BC occurrence, including aromatic amines^15^. However, the interactions between cigarette smoking and other intrinsic and environmental factors that influence BC development and progression remain poorly understood^16^.

Rates of electronic cigarette (E-cigarette) use have risen rapidly over the past decade, with a reported 900% increase in E-cigarette users among high school students between 2011 and 2015 according to the Department of Health and Human Services^17^. Despite some evidence that E-cigarette vaping may be safer than smoking traditional cigarettes^18,19^, their safety profiles remain to be fully characterized and there is a growing body of evidence highlighting the deleterious effects of vaping^20–22^. Critically, the actual composition of the E-liquids used when vaping is complex and highly variable across brands, with nicotine levels that vary anywhere from 0 to 36 mg/mL together with the presence of potentially dangerous carcinogens^23^. The inhalation of flavored E-liquid aerosols, including those free of nicotine, can induce inflammatory cytokine production and oxidative stress capable of damaging lung tissues^24^, in addition to promoting pulmonary and cardiac remodeling^25^. Exposing rats to flavored E-cigarettes can induce systemic genotoxic damage in the lungs, blood, and urine^26^, potentially further contributing to the risk of BC development. Consistently, DNA damage caused by E-cigarettes has been reported in E-cigarette-treated endothelial cells^27^. Pre-clinical and clinical studies related to BC have shown that E-cigarettes can trigger cancer-related damage to bladder tissues in mice^28^, and the urine of 92% of E-cigarette users tested positive for two chemicals, o-toluidine and 2-napthylamine, known to cause BC^29^. While new restrictions imposed by the US Food and Drug Administration (FDA) in 2020 banned the sale of many flavored E-liquids, both tobacco and menthol-flavored E-liquids remain on the market^30^. While it may take years or decades for the effects of the uptick in E-cigarette use on BC risk to be fully appreciated, given the surge in popularity of E-cigarettes and the presence of bladder carcinogens in the urine of E-cigarette users, further research is urgently needed to investigate the carcinogenic properties of different E-liquids.

EVs are small membrane-bound vesicles that are derived from the late multivesicular endosomal compartment or membrane budding. EVs can be secreted by most cell types and carry a cargo consisting of proteins, lipids, and nucleotides which can be delivered to recipient cells wherein they influence diverse physiological and pathological processes^31^. There is increasing evidence that tumor cell-derived EVs play an integral role in cancer development and progression^32^. EV-mediated cargo transfer to recipient cells affects many stages of cancer progression through communication between the cancer cells and the surrounding tumor microenvironment, such as the promotion of myofibroblast differentiation^33^, activation of proliferative and angiogenic pathways^34^, and initiation of pre-metastatic sites^35,36^. Critically, several reports from our lab and others have demonstrated the ability of BC tumor-derived EVs to initiate carcinogenesis in normal recipient urothelial cells^37–43^. While smokers reportedly exhibit increased EV release and altered EV cargo profiles in the lungs^44^, the degree to which smoking or E-cigarette use can alter the release and/or functionality of EVs derived from BC cells remains unknown.

In the context of the bladder pre-malignant field, both BCEVs and smoking or E-cigarettes can induce genotoxic stress that ultimately leads to the presence of potentially carcinogenic compounds in the bladder and drives tumorigenesis. Cigarette smoking-induced EVs and their functionality have been studied extensively in pulmonary disease^45,46^. We thus speculated that these stimuli have an opportunity to directly modulate the composition of BC and urothelial cell-derived EVs through their deleterious effects on local cells. While we and others have shown that changes in EV cargo profiles can exacerbate damage to recipient cells and support their oncogenic transformation, there remains a pressing need to clarify whether smoking or E-cigarettes can shape BC-related outcomes through a similar mechanism. To address this gap in the literature, the present study was developed to analyze the characteristics of EVs released by BC cells exposed to cigarette smoke extract (CSE) or different E-liquids, with a specific focus on the ability of these EVs to promote the malignant transformation of recipient urothelial cells consistent with a potential field cancerization effect.

## Results

### Cigarette smoke and E-liquid exposure enhance EV release from bladder cancer cells

**Figure 1:**
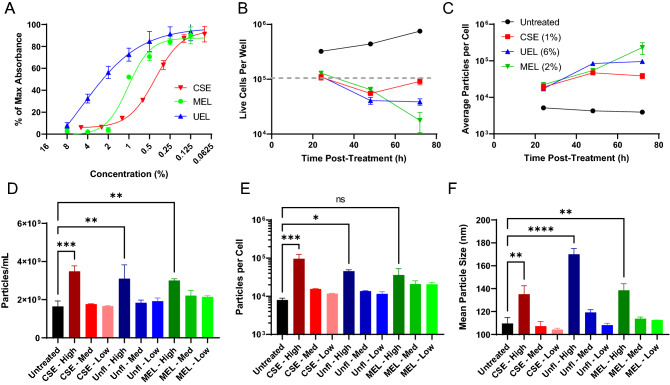
Cigarette smoke and E-liquid exposure enhance EV release from BC cells. (**A**) TCCSUP cells in 96-well plates were treated with a range of concentrations of CSE, UEL, or MEL for 24 h. An MTT assay was then used to detect cell viability. (**B,C**) TCCSUP cells in 12-well plates were treated for 24, 48, or 72 h with CSE (1%), UEL (6%), or MEL (2%). The number of live cells per well was then counted (**B**), with the gray dashed line corresponding to the number of initially plated cells. The number of EVs released per cell was further determined through a nanoparticle tracking analysis of supernatants released by these cells (**C**). (**D–F**) TCCSUP cells were treated for 24 h with low, intermediate (Med), or high concentrations of CSE, UEL, or MEL corresponding to the IC20, IC50, and IC80 concentrations of these stimulants identified in (**A**). The numbers of EVs released and the size of these EVs was then analyzed via a nanoparticle tracking analysis. CSE, cigarette smoke extract; UEL, unflavored E-liquid; MEL, methol E-liquid. Samples were analyzed in duplicate, and all results are representative of a minimum of three experiments. Data are means ± SEM. *P < 0.05, **P < 0.01, ***P < 0.001, ****P < 0.0001; one-way ANOVAs with Tukey’s post hoc test.

To begin exploring the effects of smoking-related stimuli, the grade IV human TCCSUP BC cell line was treated with a range of concentrations of prepared cigarette smoke extract (CSE) or commercially available unflavored E-liquid (UEL) or menthol-flavored E-liquid (MEL). The impact of these stimuli on cell viability was examined using an MTT assay, which revealed that all three preparations induced cell death in a dose-dependent manner (Fig. 1A), with the CSE and MEL ultimately being more toxic than UEL at a given concentration. When TCCSUP cells were treated with low concentrations of these smoking-related stimuli, progressive increases in the average number of EVs released per cell were observed over time, with a concomitant decline in cellular viability (Fig. 1B,C). To further explore the effects of these toxicants on EV release, TCCSUP cells were treated for 24 h with low, medium, or high doses respectively corresponding to the IC20, IC50, and IC80 values computed using the curves shown in Fig. 1A. In this experiment, marked increases in both total EV release and the number of EVs released per cell were observed in response to high-dose CSE, UEL, or MEL treatment (Fig. 1D,E), with more modest effects in response to lower concentrations of these stimuli. These changes in EV release dynamics were also associated with an increase in mean particle size at high concentrations, suggesting that these smoking-related stimuli may alter both the release and the characteristics of these BCEVs (Fig. 1F).

### Smoking- and E-liquid-induced BCEVs provoke inflammatory responses

**Figure 2:**
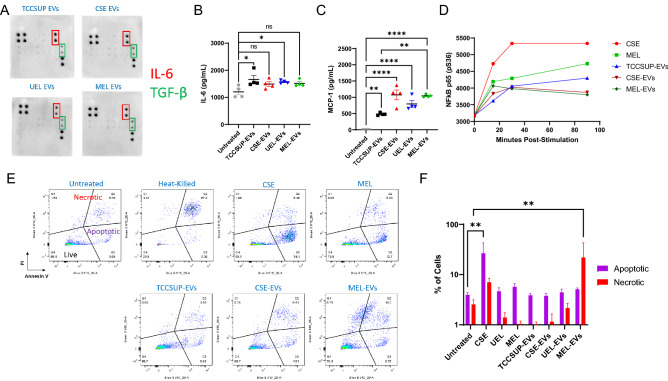
Smoking- and E-liquid-induced BCEVs provoke inflammatory responses. (**A**) Cytokine content in the indicated BCEV samples was detected using a semi-quantitative cytokine array. EVs were dissociated using 1% Triton X-100. Spots in the upper right and lower left corners are positive controls. (**B,C**) The release of IL-6 (**B**) and MCP-1 (**C**) from SV-HUC cells treated for 24 h with the indicated BCEVs (40 µg/mL) was detected using a Legendplex 13-Plex human inflammation kit by flow cytometry. (**D**) NF-κB phosphorylation was measured by flow cytometry over time in cells treated with the indicated stimuli over time. (**E,F**) The relative induction of apoptosis and necrosis in SV-HUC cells treated with the indicated BCEVs (40 µg/mL) was detected by flow cytometry using an Annexin-V/PI staining kit. Heat-killed cells were included as a positive control. All results are representative of a minimum of three experiments. Data are means ± SEM. *ns* not significant, *P < 0.05, **P < 0.01, ***P < 0.001, ****P < 0.0001; one-way ANOVAs with Tukey’s post hoc test (**B,C**) or two-way ANOVAs with Dunnett’s multiple comparisons test (**F**).

While changes in the absolute numbers of EVs released from BC cells may partially mediate any deleterious effects of smoking or E-liquid use on disease progression or recurrence, changes in EV cargo content in response to these smoking-related stimuli are also likely to play a key role in this pathological context. Prior work from our lab has demonstrated that even in the absence of caustic smoking-related stimuli, TCCSUP cell-derived EVs can promote the malignant transformation of immortalized human urothelial SV-HUC cells in a cargo protein-dependent manner^40^. Inflammation is a key driver of all stages of oncogenic progression^47^, and given that cigarette smoke or E-liquid aerosol inhalation can promote inflammatory cytokine production^24^, we next explored whether BCEVs were capable of potentiating these oncogenic inflammatory responses. A cytokine array was initially used to characterize major cargo proteins present in BCEVs released from TCCSUP cells following treatment with IC20 doses of CSE, UEL, or MEL (see “Methods”), revealing that high levels of IL-6 and TGF-β were present in all of these BCEV preparations irrespective of smoking-related stimuli (Fig. 2A). Consistently, the use of these EVs to treat SV-HUC target cells resulted in modest increases in IL-6 production as measured with a multiplexed cytokine kit (Fig. 2B), although these cells failed to upregulate other pro-inflammatory cytokines such as IL-1β or TNFα (data not shown). Notably, however, treatment of SV-HUC cells with BCEVs resulted in extremely significant increases in the release of monocyte chemotactic protein 1 (MCP-1), and this upregulation was even more pronounced when these urothelial cells were treated with CSE- or MEL-induced BCEVs (Fig. 2C). Together, these data suggest that the BCEVs released from BC cells exposed to smoking- or vaping-related toxicants are highly pro-inflammatory. In partial support of this, a time-dependent increase in the phosphorylation of the master inflammatory regulator NF-κB p65^48^ was observed in SV-HUC cells treated with these BCEV samples relative to untreated cells, although this activation was not as robust as in cells treated directly with CSE or MEL suggesting that these BCEVs do not merely deliver these toxicants to recipient urothelial cells (Fig. 2D). A flow cytometry-based analysis of the survival of SV-HUC cells treated with these BCEVs further revealed that MEL-EV exposure was sufficient to cause necrotic cell death not evident in response to other BCEV preparations (Fig. 2E,F). Together, these results suggest that in addition to triggering the increased release of EVs from BC cells, CSE and E-liquid stimulation can alter the functional properties of these BCEVs such that they can provoke distinct stimuli-specific responses in these recipient cells. While appropriate engagement of well-regulated stress responses is central to the maintenance of physiological homeostasis, the persistent induction of unresolving BCEV-induced stress, particularly in smokers or E-cigarette users, may eventually be maladaptive in a manner that is ultimately conducive to tissue damage and/or recipient cell transformation.

### Proteomic analyses of smoking- and E-liquid-induced BCEVs

**Figure 3:**
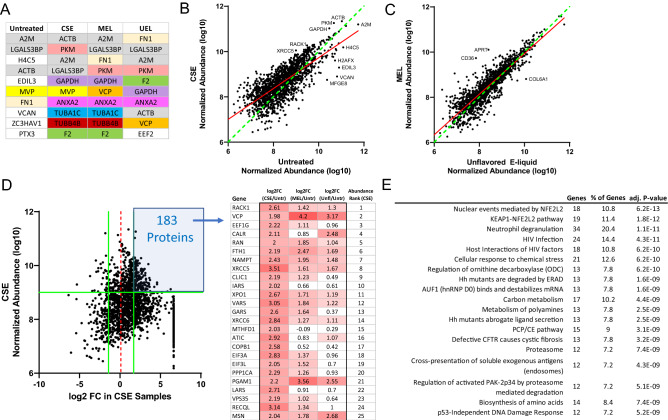
Proteomic analyses of smoking- and E-liquid-induced BCEVs. (**A**) The top 10 proteins in EVs from the indicated TCCSUP cell treatment groups, ordered based on relative abundance. (B, C) Comparison of the relative abundance of all proteins detected in both CSE and untreated TCCSUP cell EV samples (**B**) and UEL and MEL EV samples (**C**). Data represent log10-transformed normalized abundance values. (**D**) Approach to the selection of the top enriched proteins in EVs derived from CSE-treated TCCSUP cells (log 2 FC > 1.5, normalized abundance > 10^9^). The top enriched genes are shown ordered based on their overall abundance in CSE-EVs, with corresponding fold-change values for these proteins in the EVs from each of the three indicated TCCSUP EV groups relative to EVs from untreated TCCSUP cells. (**E**) KEGG and Reactome pathway enrichment analysis results for the 183 proteins highlighted in (**D**), performed with the DAVID database.

An unbiased LC–MS/MS approach was next used to analyze samples of TCCSUP-EVs, CSE-EVs, UEL-EVs, and MEL-EVs in order to characterize the cargo content present therein in hopes of exploring the links between these cargo profiles and the functional properties of these EVs. When focusing on the top 10 most abundant cargo proteins in these EVs (Fig. 3A), all three smoking-related EVs were largely distinct from TCCSUP-EVs, with the cargo profiles of CSE-EVs and MEL-EVs being particularly similar. The cargo profile of untreated TCCSUP-EVs also differed from that of CSE-EVs when examining the overall cargo proteome (Fig. 3B), with particularly divergent protein levels for low-abundance cargos. In contrast, the cargo protein profiles of UEL-EVs and MEL-EVs were largely similar to one another (Fig. 3C). By using selected criteria (log2 FC > 1.5, Normalized abundance > 10^9^) to focus more specifically on highly abundant cargos enriched in these smoking-related BCEVs, we identified a list of 183 cargo proteins of interest (Fig. 3D). Many of these proteins were enriched in all three smoking-related EV subtypes relative to untreated TCCSUP-EVs, while several were enriched specifically in CSE-EVs and MEL-EVs. Pathway enrichment analyses of these 183 proteins indicated that they were closely associated with the oxidative stress-related “nuclear events mediated by NFE2L2” and “NEAP1-NFE2L2” pathways (Fig. 3E). Notably, these proteins were significantly enriched in the “p53-independent DNA damage response” pathway, suggesting the potential ability of these EVs to sustain cell survival under conditions of genotoxic stress, providing additional opportunities for the accumulation of mutations and consequent malignant transformation.

### Smoking- and E-liquid-induced BCEVs are genotoxic and promote oxidative stress

**Figure 4:**
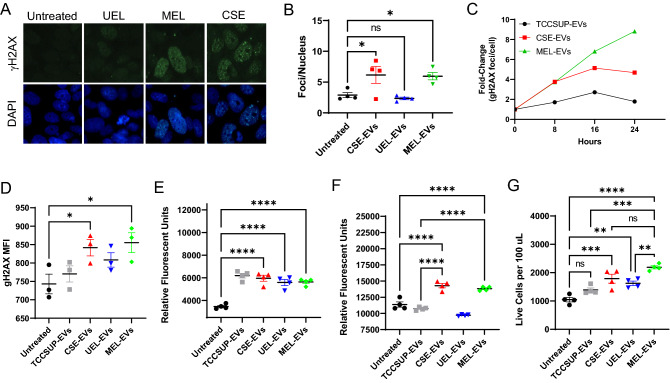
Smoking- and E-liquid-induced BCEVs are genotoxic and promote oxidative stress. (**A,B**) SV-HUC cells were left untreated or were treated with the indicated BCEVs (40 µg/mL) for 18 h, after which cells were stained for γH2AX foci (green) and counterstained with DAPI (blue). Representative fields of view are shown in (**A**), with corresponding quantification in (**B**). (**C**) Cells were treated as in (**A**) for 0, 8, 16, or 24 h, after which γH2AX foci were counted via immunofluorescent microscopy. (**D**) SV-HUC cells were treated for 24 h with CSE (1%), MEL (2%), or the indicated BCEVs, after which p-γH2AX was detected by phosphoprotein flow cytometry. (**E–G**) SV-HUC cells were treated for 24 h with the indicated BCEVs, after which intracellular ROS levels were analyzed via CellROX staining (**E**), while H_2_O_2_ levels in cell culture supernatants were detected via Amplex assay (**F**), and live cells in each well were counted (**G**). All results are representative of a minimum of three experiments. Data are means ± SEM. *ns* not significant, *P < 0.05, **P < 0.01, ***P < 0.001, ****P < 0.0001; one-way ANOVAs with Tukey’s post hoc test.

Smoking is a major risk factor for BC development, and work from our laboratory has previously demonstrated the ability of BCEVs to contribute to genotoxic stress in recipient urothelial cells, thereby contributing to their malignant transformation^40^. Given this prior evidence and the above data demonstrating that the proteins enriched in CSE-EVs and MEL-EVs are associated with genotoxic and oxidative stress pathways, we next sought to explore whether CSE- or E-liquid-induced BCEV treatment would contribute to higher levels of recipient cell stress. The direct treatment of SV-HUC cells with CSE-EVs or MEL-EVs, but not UEL-EVs, was sufficient to induce the formation of γH2AX foci consistent with DNA damage after 18 h (Fig. 4A,B). Strikingly, time-dependent changes in γH2AX foci formation were observed over a 24 h treatment period such that both CSE-EVs and TCCSUP-EVs induced double-stranded break formation in the DNA of recipient SV-HUC cells; this damage peaked after 16 h before foci numbers declined, consistent with the induction of DNA repair responses (Fig. 4C). In contrast, MEL-EVs induced higher levels of DNA damage in these recipient cells, and this damage had not begun to resolve within the 24 h experimental period consistent with ongoing persistent genotoxic stress. Consistently, flow cytometry analyses revealed higher levels of γH2AX foci in SV-HUC cells that had been treated with CSE-EVs and MEL-EVs for 24 h (Fig. 4D). Analyses of ROS production revealed that while treatment with all four BCEV preparations increased intracellular ROS levels as measured using the CellROX reagent (Fig. 4E), only treatment with CSE-EVs or MEL-EVs was sufficient to increase the release of H_2_O_2_ into cell culture supernatants (Fig. 4F). Unexpectedly, CSE-EV, UEL-EV, and MEL-EV treatments were all associated with a significant increase in recipient cell proliferation after 24 h, with this effect being most pronounced following MEL-EV treatment (Fig. 4G). Together, these results suggest that the EVs released from BC cells exposed to CSE of E-liquids can induce DNA damage and oxidative stress in recipient cells while simultaneously serving as mitogenic stimuli that promote cell proliferation, potentially providing more opportunities for malignant transformation to occur.

### E-liquid-induced BCEVs promote the malignant transformation of SV-HUC cells

**Figure 5:**
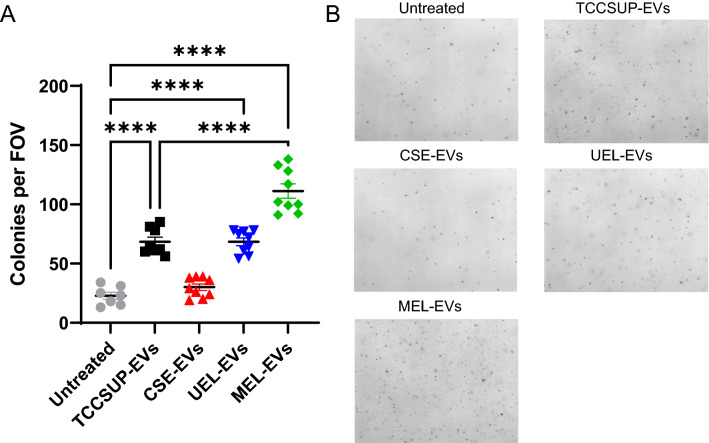
E-liquid-induced BCEVs promote the malignant transformation of SV-HUC cells. SV-HUC cells in 24-well plates were treated twice per week with the indicated BCEVs (20 µg/mL) for 12 weeks. After a 4-week recovery period, a soft agar colony formation assay was performed using these cells (6 wells/treatment condition), with five random fields of view per well being imaged after 6 weeks. The number of colonies in each well was analyzed across five random FOVs in each well (**A**), with representative FOVs being shown in (**B**). Data are means ± SEM. ****P < 0.0001, one-way ANOVA with Tukey’s post hoc test.

To test the hypothesis that smoking- or E-liquid-induced BCEVs can induce urothelial cell transformation more readily than EVs isolated from untreated TCCSUP cells, we conducted a long-term treatment assay. SV-HUC cells were repeatedly exposed to TCCSUP-EVs, CSE-EVs, UEL-EVs, or MEL-EVs for 12 weeks, after which these cells were allowed to grow for an additional 4 weeks without further treatment to permit the stabilization of any genetic changes present within these populations. Then, a soft agar colony formation assay was conducted to test for anchorage-independent cell growth. In line with our prior work, TCCSUP-EVs significantly increased the number of colonies observed in the soft agar matrix (Fig. 5). Unexpectedly, CSE-EVs were associated with less robust colony formation suggestive of a net reduction in malignant transformation, potentially as a byproduct of the observation that these CSE-EV-treated urothelial cells grew more slowly and exhibited poorer viability than all other tested SV-HUC cells (data not shown). While UEL-EVs did not have any pronounced impact on urothelial cell transformation, MEL-EV treatment significantly increased colony formation relative to TCCSUP-EV treatment (Fig. 5). These results are consistent with a model wherein MEL-EVs are able to more readily enable SV-HUC cells to proliferate and grow in an anchorage-independent manner, potentially contributing to tumor dissemination.

## Discussion

BC is a highly prevalent disease that continues to impose high levels of physical, psychological, and economic stress on affected patients^1–3,5^. While smoking is the best-characterized risk factor associated with BC^10–14^, whether the recent surge in E-cigarette use will similarly contribute to BC development remains to be established, although the presence of bladder carcinogens in the urine of E-cigarette users does support this possibility^29^. In this study, we explored the ability of EVs derived from advanced BC cells exposed to CSE or E-liquid preparations to impact the physiology of recipient urothelial cells and to contribute to their malignant transformation, while also characterizing the protein cargo profiles of these different populations of BCEVs. These experiments revealed that CSE or E-liquid exposure profoundly altered the proteins present within these BCEVs, with a concomitant increase in the ability of CSE-EVs and MEL-EVs in particular to provoke inflammation, oxidative stress, and DNA damage in recipient urothelial cells. Importantly, prolonged MEL-EV treatment also contributed to the enhanced ability of these SV-HUC recipient cells to engage in anchorage-independent growth consistent with malignant transformation. In prior work from our lab, we have demonstrated that the oncogenic potential of BCEVs is not restricted to a single BC cell line, with EVs from both TCCSUP (Grade IV) and T24 (Grade III) BC cells promoting SV-HUC cell transformation^40^. In that same study, we also confirmed that the enhanced anchorage-independent growth of SV-HUC cells in a soft agar colony forming assay was directly associated with the enhanced ability of these cells to form tumors in a murine xenograft model system, and we thus expect these observations to hold true in the present experimental context. Together, these data reaffirm the important status of BCEVs as potential mediators of field cancerization that may contribute to BC recurrence or progression, while also highlighting the fact that menthol-flavored E-liquid use may be associated with even higher levels of clinical risk in susceptible individuals who use this product, although further preclinical and clinical validation will be essential to test this possibility.

While these results offer strong in vitro preclinical evidence supporting a model wherein menthol-flavored E-liquid exposure can ultimately provoke the release of EVs from BC cells capable of promoting urothelial cell transformation, they also highlight many areas that warrant further study. Strikingly, while MEL-EVs significantly enhanced urothelial cell transformation on a per-EV basis, the same was not true for UEL-EVs (Fig. 5), although the fact that high levels of UEL exposure did enhance EV release from BC cells (Fig. 1) would nonetheless support the ability of even UEL to drive EV-mediated field cancerization in BC patients. Given that the protein cargo profiles of UEL-EVs and MEL-EVs were nearly identical to one another (Fig. 3C), this may suggest that other EV cargos such as lipids or microRNAs may also contribute to the enhanced genotoxicity of MEL-EVs. The exposure of mice to E-cigarette aerosols induces bladder toxicity^28^, and bladder carcinogens have been detected in the urine of 92% of E-cigarette users consistent with the potential carcinogenic effects of both UEL and MEL preparations^29^. Moreover, menthol itself is a toxicant, and prior in vitro evidence suggests that menthol-flavored E-liquids have a lower pH than other E-liquids and induce high levels of cell death in a range of model systems^49,50^, in line with their greater cytotoxic potency relative to UEL preparations in this study. The in vivo toxicity and potential bladder carcinogenicity of menthol E-liquids is further supported by evidence that menthol E-liquid aerosols contain higher levels of formaldehyde and acrolein, which can promote DNA adduct formation and p53 mutation^51,52^, relative to other E-liquids, with a concomitant increase in urinary concentrations of the acrolein metabolite 3-hydroxypropyl mercapturic acid (3-HPMA)^53^. Whether these MEL-derived metabolites directly influence BCEV composition or are capable of being delivered to urothelial cells within these EVs will require further investigation. Additional comparisons of the short- and long-term effects of MEL and MEL-EVs, both alone and in combination, on urothelial cells are also warranted to fully clarify which of the observed effects are specifically tied to the oncogenic properties of these MEL-EVs.

Our observation that CSE-EVs failed to induce malignant urothelial cell transformation in long-term assays is surprising, particularly given that these BCEVs promoted inflammatory responses, DNA damage, and ROS production to a degree similar to that observed for MEL-EVs in short-term assays. The status of cigarette smoking as a risk factor for BC is firmly established^10–14^, and smoking has been shown to be associated with shifts in EV cargo profiles associated with non-small cell lung cancer akin to the proteomic changes observed in this study^44^. While we did not observe any high levels of acute toxicity associated with short-term CSE-EV exposure in this study, we did find that over the course of the 16-week long-term treatment period, CSE-EV-treated SV-HUC cells tended to grow increasingly slowly, ultimately exhibiting near-total growth arrest 4 weeks after the completion of the long-term colony formation assay (data not shown). This likely accounts for the poor performance of these cells in the soft agar colony formation assay. One possibility is that this impaired growth was the consequence of the accumulation of genotoxic damage and other forms of cell stress at rates that exceeded the capacity of these cells to mitigate such stress, thereby inducing senescence and eventual death. However, the etiological basis for this unexpected finding remains to be clarified in future studies.

The EV cargo analyses performed herein revealed the enrichment of several potentially consequential proteins in BCEVs from CSE- or MEL-treated cells. Phosphoglycerate mutase 1 (PGAM1) was enriched at higher levels in MEL-EVs as compared to both CSE-EVs and UEL-EVs. Intriguingly, in previous reports researchers have specifically identified PGAM1 as a pro-oncogenic protein that is upregulated in BC cells, supporting aerobic glycolysis and tumor cell growth^54^. Whether the BCEV-mediated delivery of PGAM1 to recipient urothelial cells contributes to metabolic changes conducive to malignant transformation of the superior mitogenic effects of MEL-EVs (Fig. 4G) may thus be an interesting topic for future study. Vasolin-containing protein (VCP) was similarly enriched in MEL-EVs. In addition to its reported association with recurrent follicular thyroid cancer and hepatocellular carcinoma^55,56^, Kigas et al. found that VCP was upregulated in invasive BC cells and that inhibiting this protein increased the sensitivity of these cells to ionizing radiation^57^. The MEL-EV-mediated delivery of VCP to recipient urothelial cells may similarly render them more resistant to other forms of genotoxic stress induced upon BCEV exposure, ultimately better equipping these cells to undergo malignant transformation. CSE-EVs and MEL-EVs also both exhibited increased abundance of the X-ray cross-complementing (XRCC) protein family members XRCC5 and XRCC6, which function as a heterodimeric complex to facilitate double-stranded DNA break repair^58^. Both XRCC5 and XRCC6 are reportedly upregulated in BC and associated with disease progression^59^, though whether they can contribute to a field cancerization effect by better enabling urothelial cells to repair DNA damage induced in response to ROS production or other factors has yet to be established. Accordingly, further research efforts are necessary to characterize the functional role of these and other individual MEL-enriched BCEV cargo proteins in the process of the EV-mediated urothelial cell transformation.

When we analyzed the inflammatory cytokines secreted by BCEV-treated SV-HUC cells, we unexpectedly observed the production of high levels of MCP-1 (also known as CCL2) from these cells following treatment with TCCSUP-derived BCEVs, and this upregulation was even more pronounced upon CSE-EV or MEL-EV treatment (Fig. 2C). CCL2 is a potent pro-inflammatory chemokine that serves as a chemoattractant for myeloid cells and has been extensively studied as a predictor and potential driver of tumor cell growth and metastasis^60,61^. Whether autocrine signaling through the CCL2 receptor protein CCR2 occurs in transformed SV-HUC cells remains to be established, although other groups have reported non-transformed SV-HUC cells to be unresponsive to recombinant CCL2 whereas this chemokine can drive the migration and invasion of BC cells derived from more advanced tumors^62^. However, there have been conflicting reports regarding the role of CCL2 in BC. The majority of studies have linked this chemokine with malignant processes including lymphatic metastasis^63^, angiogenesis^64^, and epithelial-mesenchymal transition^65^, and tumor cell CCL2 expression has been identified as an independent predictor of negative BC patient prognostic outcomes^66^. Additional research will help clarify whether the BCEV-driven elicitation of robust CCL2 production plays any role in BC recurrence or primary BC tumor progression, with smoking or E-cigarette use potentially exacerbating any emergent oncogenic phenotypes.

In conclusion, our results suggest that BCEVs can promote inflammation, oxidative stress, and DNA damage in non-malignant urothelial cells, increasing their risk of subsequent carcinogenesis. This process can be further exacerbated when BC cells are exposed to certain E-liquid preparations or CSE. In vivo*,* urothelial cells in the bladder of smokers or E-cigarette users with BC would be simultaneously exposed to CSE- or E-liquid-derived carcinogenic compounds and related BCEVs, which we expect to further compound the BCEV-associated risk of malignant transformation observed in this study. The degree to which this model holds true in a clinical setting is an important focus for future research. Additional studies examining whether these smoking- and E-cigarette-related preparations can alter the EV cargo profiles of non-transformed urothelial cells in a pro-oncogenic manner should be conducted in order to gain more comprehensive insight into the risk profiles associated with these toxicants. In addition, the functional characterization of EVs from long-term smokers and E-cigarette users with and without BC will be central to clarifying the public health implications of these results. However, given that the true oncogenic risks of long-term E-cigarette use in human populations may not be apparent for decades given the relatively recent advent of these cigarette alternatives such that these analyses cannot be readily performed at present, we believe that our findings highlight the potential risks of E-cigarette use, providing a strong foundation for future studies focused on the role that EVs play as facilitators of field cancerization and tumor recurrence in this context and more broadly.

## Methods

### Cell culture and stimulation

TCCSUP and SV-HUC cells were obtained from the American Type Culture Collection (ATCC, USA), and were respectively cultured in DMEM and Ham’s F-12K medium (Gibco, USA). TCCSUP cell culture medium was supplemented with 10% fetal bovine essence (FBE; Gibco, USA), non-essential amino acids, and penicillin/streptomycin, while SV-HUC culture medium was supplemented with 10% fetal bovine serum (FBS) and penicillin/streptomycin. All culture reagents were from Thermo Fisher Scientific (USA) unless otherwise indicated. Cells were routinely cultured in a standard humidified 37 °C incubator under 5% CO_2_.

For analyses of EV production, cells were plated in 12- or 96-well culture plates and allowed to adhere overnight, after which media was exchanged for serum-free media supplemented with the indicated concentrations of menthol-flavored E-liquid (MEL; Vista Vapors, USA), unflavored E-liquid (UEL; Vista Vapors, USA), or cigarette smoke extract (CSE). CSE was prepared as previously described using impingers by vigorous bubbling from one 3R4F cigarette into 10 mL of serum free-culture medium^67^. At appropriate time points, cells or culture supernatants were used for downstream analyses detailed below.

### MTT assay

At 24, 48, or 72 h following the start of treatment with smoking-related stimuli as detailed above, cell viability was examined using an MTT assay according to standard protocols^40^. Briefly, 10 μl of 12 mM MTT solution (Invitrogen, USA) was added per well. Following a 2 h incubation at 37 °C, culture medium was removed, and 75 μl of DMSO was added per well, after which absorbance at 540 nm was measured with a Synergy H1 microplate reader (BioTek Instruments, Inc.).

### Nanoparticle tracking analysis

Cell culture supernatants were harvested from treated cells at appropriate time points, centrifuged twice (15,500×*g*, 30 min) to remove cellular debris, and transferred into Eppendorf tubes. Samples were then analyzed using a Nanosight NS3000 instrument (Malvern Panalytical, UK). Particle data for each sample was collected in three 30 s video files. Camera level and detection threshold values remained constant for all analyses to ensure consistency.

### Extracellular vesicle preparation

EVs from TCCSUP cells that had been treated with UEL (6%), MEL (2%), or CSE (1%) were prepared using CELLine 1000AD Bioreactor flasks (Wheaton, USA). Briefly, each flask was seeded with 25 × 10^6^ TCCSUP cells in complete culture medium and allowed to incubate for 14 days. Medium in the cell compartment was then exchanged for an equivalent volume of medium supplemented with the selected concentration of UEL, MEL, or CSE, and cells were incubated overnight. Medium was then exchanged for EV-depleted complete culture medium, and cells were incubated for 3 days, after which medium was collected from each flask and stored at −80 °C. After a 4-day rest period during which cells were cultured in complete culture medium, cells were again stimulated, and this process was repeated weekly for a maximum of 6 months.

EVs were isolated from these cell culture supernatants using a standard approach. Briefly, thawed samples were centrifuged once at 400×*g* (10 min, 4 °C) and twice at 15,500×*g* (30 min, 4 °C) to remove cellular debris. Samples were then subject to two rounds of ultracentrifugation (44,000×*g*, 70 min, 4 °C), and the EV-containing pellets were resuspended in a small volume of Dulbecco’s Phosphate Buffered Saline (DPBS; Thermo Fisher Scientific, USA). Total protein concentrations in these samples were measured via a Micro BCA assay (Thermo Fisher Scientific, USA), and EVs were stored at −80 °C in small aliquots for subsequent use.

### EV cytokine array analysis

The protein content in EV samples of interest was analyzed using a protein microarray kit (RayBiotech Inc., USA) based on provided directions. Briefly, EVs were dissociated by incubating 40 µg of EVs with 1% Triton X-100 for 1 h at room temperature with occasional shaking. Samples were then diluted with DPBS to a final volume of 1000 µL containing protease inhibitors (Pierce, USA) and incubated overnight with blocked cytokine array membranes at 4 °C with gentle rocking.

### Legendplex cytokine assay

Cytokine levels in supernatants released from SV-HUC cells at 24 h post-stimulation were measured using a LEGENDplex™ Human Inflammation Panel 1 (13-plex) kit (BioLegend, USA) based on provided directions. Samples were analyzed in duplicate with an LSR II flow cytometer (BD Biosciences, USA), and the resultant data were evaluated using the provided LegendPlex Data Analysis Software Suite (BioLegend).

### Flow cytometry

For analyses of apoptotic cell death, an Dead Cell Apoptosis Kit with Annexin V for Flow Cytometry (Thermo Fisher Scientific, USA) was used based on provided directions. Briefly, cells were treated for 24 h with the indicated BCEVs, after which they were harvested, stained with Annexin V and propodium iodide (PI), and analyzed via flow cytometry (LSR II, BD Biosciences, USA). For phospho-protein staining, cells were treated with the indicated smoking-related stimuli for an appropriate amount of time, after which they were resuspended in 100 µL of culture medium and fixed with an equal volume of BD Cytofix fixation buffer for 15 min at 37 °C, resuspended in 150 µL of BD Cytoperm Solution III for 30 min on ice, and resuspended in PBS containing 2% FBS and PE Mouse Anti-H2AX (pS139) (1:50) or PE Mouse anti-NF-κB p65 (pS529) (1:50) for 20 min at room temperature. Cells were then washed and analyzed via flow cytometry. All flow cytometry data were analyzed using FlowJo v 10.8.1.

### Reactive oxygen species assays

After treatment for 24 h with the indicated BCEVs, SV-HUC cell culture supernatants were harvested and the relative levels of H_2_O_2_ present therein were detected using an Amplex™ Red Hydrogen Peroxide/Peroxidase Assay Kit (Invitrogen, USA) based on provided directions. In parallel, the treated cells were stained with the CellROX Deep Red reagent (5 µM) for 30 min at 37 °C, washed twice with DPBS, and analyzed via flow cytometry (LSR II, BD Biosciences, USA).

### Immunofluorescent staining

SV-HUC cells were treated with BCEVs (40 µg/mL) for 18 h in chamber slides. Cells were then washed with PBS and fixed in 1% ultrapure formaldehyde (catalog no. 04018, Polysciences) in PBS for 20 min at room temperature. After additional washing with PBS, cells were treated with methanol for 10 min at −20 °C, air dried, and permeabilized with acetone (catalog no. A18-1, Fisher Scientific) for 1 min at room temperature. Cells were then blocked with 5% normal swine serum for 1 h at room temperature prior to overnight primary antibody incubation at 4 °C using anti-γH2AX (1:400; catalog no. 05-636, Millipore) and secondary staining with Alexa Fluor 488 F(ab’)2 fragment IgG (catalog no. A11017, Invitrogen). DAPI was used for nuclear counterstaining. γH2AX foci and nuclei were counted using an ImageJ plugin (EZ foci PZ).

### Proteomic analyses

The proteins present within BCEVs were analyzed using a quantitative LC–MS/MS approach detailed in our recent study^68^. All pathway enrichment analyses of differentially abundant proteins in EV samples were performed using the DAVID bioinformatics database (https://david.ncifcrf.gov/).

### Soft agar colony forming assay

The ability of BCEVs derived from TCCSUP cells treated with UEL, MEL, or CSE to induce the transformation of SV-HUC cells was assessed by subjecting these cells to long-term BCEV treatment and then performing a soft agar colony forming assay, in vitro tumorigenic assay, to test for anchorage-independent growth^40^. Briefly, SV-HUC cells were plated in 24-well plates (2 × 10^4^/well) and treated twice per week with EV-depleted complete culture medium containing 20 µg/mL of BCEVs isolated from TCCSUP cells that were untreated or were treated with UEL, MEL, or CSE (8 replicate wells per treatment condition). Cells were passaged when 80% confluent. After 12 weeks, treatment was discontinued and cells were cultured for 4 weeks without exogenous stimulation.

To prepare the soft agar colony forming assay, wells of a six-well plate were filled with 2 mL of 0.8% Noble agar (Sigma-Aldrich, USA) in serum-free F-12K medium. After the medium had solidified, BCEV-treated SV-HUC cells were suspended at 1 × 10^5^ cells/mL in medium containing 0.4% Noble agar and added atop the established 0.8% agarose layer (6 wells per treatment condition). After the cell-containing medium had solidified, 1.5 mL of serum-free culture medium was added to each well. Medium was replaced once per week for 6 weeks. Colonies were then photographed in five random fields of view (FOV) per well, and the ImageJ software was used to count the number of colonies in each FOV.

### Statistical analysis

GraphPad Prism 9.2.0 (GraphPad Software, LLC, USA) was used to conduct all statistical analyses in this study. P < 0.05 was the threshold of statistical significance, and details regarding the numbers of experimental replicates and the statistical tests employed are provided in the corresponding Figure Legend text.

## Data Availability

The datasets generated during and/or analyzed during the current study are available from the corresponding author on reasonable request.
